# X-ray characterization of mesophases of human telomeric G-quadruplexes and other DNA analogues

**DOI:** 10.1038/srep27079

**Published:** 2016-06-02

**Authors:** Selcuk Yasar, Jacob B. Schimelman, M. Alphan Aksoyoglu, Nicole F. Steinmetz, Roger H. French, V. Adrian Parsegian, Rudolf Podgornik

**Affiliations:** 1 Department of Physics, University of Massachusetts, Amherst, MA 01003, United States; 2 Department of Macromolecular Science and Engineering, Case Western Reserve University, 10900 Euclid Avenue, Cleveland, Ohio 44106, United States; 3 Department of Biomedical Engineering, Case Western Reserve University, 10900 Euclid Avenue, Cleveland, Ohio 44106, United States; 4 Department of Radiology, Case Western Reserve University, 10900 Euclid Avenue, Cleveland, Ohio 44106, United States; 5 Department of Materials Science and Engineering, Case Western Reserve University, 10900 Euclid Avenue, Cleveland, Ohio 44106, United States; 6 Comprehensive Cancer Center, Division of General Medical Sciences and Oncology, Case Western Reserve University, 10900 Euclid Avenue,Cleveland, Ohio 44106, United States; 7 Department of Physics, Case Western Reserve University, 10900 Euclid Avenue, Cleveland, Ohio 44106, United States; 8 Department of Theoretical Physics, J. Stefan Institute, SI-1000 Ljubljana, Slovenia; 9 Department of Physics, Faculty of Mathematics and Physics, University of Ljubljana, SI-1000 Ljubljana, Slovenia

## Abstract

Observed in the folds of guanine-rich oligonucleotides, non-canonical G-quadruplex structures are based on G-quartets formed by hydrogen bonding and cation-coordination of guanosines. In dilute 5′-guanosine monophosphate (GMP) solutions, G-quartets form by the self-assembly of four GMP nucleotides. We use x-ray diffraction to characterize the columnar liquid-crystalline mesophases in concentrated solutions of various model G-quadruplexes. We then probe the transitions between mesophases by varying the PEG solution osmotic pressure, thus mimicking *in vivo* molecular crowding conditions. Using the *GMP-quadruplex*, built by the stacking of G-quartets with no covalent linking between them, as the baseline, we report the liquid-crystalline phase behaviors of two other related G-quadruplexes: (i) the intramolecular parallel-stranded G-quadruplex formed by the 22-mer four-repeat human telomeric sequence AG_3_(TTAG_3_)_3_ and (ii) the intermolecular parallel-stranded G-quadruplex formed by the TG_4_T oligonucleotides. Finally, we compare the mesophases of the G-quadruplexes, under PEG-induced crowding conditions, with the corresponding mesophases of the canonical duplex and triplex DNA analogues.

Human telomeric sequences AG_3_(TTAG_3_)_*n*_ can form four-stranded G-quadruplexes^1^ by folding on themselves and matching the G_3_ segments, enabling the formation of G-quartets^2^. Polymorphic G-quadruplex structures have been implicated in several biological processes, such as telomere formation in aging and in disease development^3^. In particular, G-quadruplex conformations of the four-repeat human telomere AG_3_(TTAG_3_)_3_ in the presence of K^+^ ions have been an important research focus^1^,^4^: The parallel-stranded conformation is observed in the crystalline state^5^ and in K^+^ solutions^6^,^7^ in the presence of polyethylene glycol (PEG). G-quadruplexes formed by the four-repeat human telomere have been shown^8^ to be thermally more stable than the structures formed by the longer telomeric sequences, implying that the four-repeat telomere is the likely candidate for G-quadruplex formation in human cells^9^. Here, the parallel-stranded intramolecular G-quadruplex formed by AG_3_(TTAG_3_)_3_ will be referred to as the *22-mer HT-quadruplex*. This quadruplex^5^ (PDB: 1KF1) contains a central core formed by the stacking of three G-quartets supported by four parallel sugar-phosphate strands and TTA linkers that connect the adjacent strands by forming side-loops (Fig. 1a,b). Mimicking molecular crowding conditions *in vivo*, that critically affect the structure of G-quadruplexes^10^, by systematically varying the osmotic pressure exerted by the bathing PEG solutions and combining it with the x-ray structural probe in an osmotic stress experiment^11^, we examine the liquid-crystalline phase behavior of the *22-mer HT-quadruplex* in order to assess its likely state under similar conditions in the cellular environment.

The G-quartet is a planar structure formed by the cyclic arrangement of four hydrogen-bonded guanine residues. We first examine the liquid-crystalline phases of the G-quartets that are made of four GMP monomers^12^ (Fig. 1c). The columnar structure built by the stacking of such G-quartets (abbreviated *GMP-quadruplex*) is a simple model of biologically relevant G-quadruplexes. Helical stacking of G-quartets (Fig. 1d) in the crystalline state was proposed based on fiber diffraction^13^. Formation of columnar structures was detected also in the non-crystalline GMP solutions in the presence of K^+^ ions^14^. By varying the osmotic pressure of the solution set by the concentration of PEG, we investigate the first-order transition from a loosely linked and disordered G-quartet column formed in K^+^ solution to the highly-ordered *GMP-quadruplex* driven by the osmotic pressure changes in the regime mimicking the biologically relevant molecular crowding conditions. This osmotic-pressure-induced change in order along the columnar axis consequently permits tighter packing of the *GMP-quadruplex* array between the columns, with progressively longer-ranged hexagonal order in the plane perpendicular to the columnar axis.

The columnar liquid-crystalline mesophases of planar disc-shaped structures are known and understood^15^. In these phases, disc-shaped structures are stacked on top of each other and form columns, which in turn self-assemble into arrays. As with other columnar assemblies^16^, the nature of intra- and intercolumnar ordering can vary, depending on solution conditions. The disordered columnar phases (Φ_*dc*_) exhibit fluid-like positional intracolumnar order, while in the ordered columnar phases (Φ_*oc*_) there is long-range positional order within each column. This leads to column-column positional and orientational correlations and consequently to long-range intercolumnar order^16^,^17^. In this respect, the mesophase transition of *GMP-quadruplex* is similar to the Φ_*dc*_ → Φ_*oc*_ transitions observed in the columnar aggregates of other disc-shaped structures built from the molecules with aromatic rings^15^,^17^,^18^.

Recent experiments demonstrated that the formation of higher-order G-quadruplex motifs in the human telomere is sensitive to the phase in the cell-cycle^9^, which, on the other hand, is related to macromolecular crowding, that can fine-tune the gene circuit response^19^. Thus, the Φ_*dc*_ → Φ_*oc*_ transition of the *GMP-quadruplex* induced by changing molecular-crowding conditions is relevant for the higher-order G-quadruplex structure formation built from the *22-mer HT-quadruplex* repeats^5^,^8^. In this view, we also examined the columnar assemblies and the mesophase behaviors of (i) the *22-mer HT-quadruplex* and (ii) the intermolecular parallel-stranded G-quadruplex formed by four TG_4_T oligonucleotides^20^ (PDB:244D). The latter (abbreviated *TG*_4_*T-quadruplex*) is shown in Fig. 2a. This quadruplex was selected because of its resemblance to the *22-mer HT-quadruplex*. In both structures, parallel sugar-phosphate backbones interconnect the stacked G-quartets. Contrary to the *22-mer HT-quadruplex*, the *TG*_4_*T-quadruplex* is also missing the flexible side loops.

The Φ_*dc*_ → Φ_*oc*_ transition of the *GMP-quadruplex* is also analogous to the mesophase transitions in duplex (Fig. 2b) and triplex (Fig. 2c) DNA arrays under similar crowding conditions. For duplexes, this transition is described as the cholesteric-columnar hexagonal transition for short DNA^21^ and as the cholesteric-hexatic transition for long DNA^22^,^23^. While the columnar hexagonal phase of the DNA triplexes has been observed before^24^, the triplex mesophase transition was not measured. The changes observed in the x-ray diffraction patterns at the mesophase transitions of the duplex, triplex, and G-quadruplex are essentially similar. At these transitions, both the intra- and the intercolumnar order change significantly and abruptly. We discuss the mesophase transitions of the analogue DNA structures with or without sugar-phosphate backbones and with different numbers of bases contributing to the stacking units (i.e., base-pair for duplex, base-triplet for triplex, and G-quartet for G-quadruplex). We then assess the relevance of these transitions for the observed mesophase behaviors.

Finally, *22-mer HT-quadruplex* has a propeller-like shape^5^ though not as pronounced as when *22-mer HT-quadruplex* blocks are stacked in a column with TTA *linkers* between them^1^,^5^. In this particular model for the columnar stacking of G-quadruplex blocks (that are formed by four-repeat telomeric sequences), TTA segments connect the consecutive blocks that in turn would form an ordered G-quadruplex column with quasi-continuous helical characteristics. Here, we investigate the columnar mesophase behavior of *22-mer HT-quadruplex* blocks when they are *unlinked*. These are similar to the unlinked very-short duplex DNA fragments^25^, except that the strong stacking interactions between the exposed hydrophobic cores of the duplex DNA fragments^25^ are missing in the *22-mer HT-quadruplex* case. If anything, because of the relatively weak end-to-end stacking interactions, the *22-mer HT-quadruplex* blocks could be more prone to repel each other then to attract. This would force the columns of *22-mer HT-quadruplex* blocks to be highly disordered.

## Results

Under sufficient molecular crowding, as mimicked by the PEG-induced osmotic pressure (see *Methods*), duplex and quadruplex (*GMP-quadruplex, TG*_4_*T-quadruplex*, and *22-mer HT-quadruplex*) DNA structures self-assemble into stable aggregates in the presence of K^+^ ions without any other ions being added. These aggregates are transferred into PEG solutions of various concentrations for equilibration against known *external* osmotic pressures. PEG (molecular weight 8000 Daltons) is excluded from the DNA arrays during equilibration. Temperature-dependent osmotic pressures produced by the solutions of PEG at various concentrations are from ref. 11. Unless otherwise stated, all the measurements are at [KCl] = 0.3 M.

The osmotic pressures required for inducing DNA-analogue mesophase transitions strongly depend on solution ionic conditions. At [K^+^] = 0.3 M, in the absence of any multivalent salts, the transition osmotic pressures for duplex and *GMP-quadruplex* are about the same, varying nearly from 6 to 8 atm (corresponding to from ≈19 to ≈22 wt% PEG 8000 concentration at 20 °C). Increasing K^+^ concentration (under fixed external pressure) results in compression in the arrays of duplexes and G-quadruplexes. This observation can be explained as the screening of the electrostatic interactions between intercolumnar phosphate charges.

In this section, we describe and present the experimental data systematically, by giving the priority to G-quadruplex DNA structures (*GMP-quadruplex, TG*_4_*T-quadruplex*, and *22-mer HT-quadruplex*) and by using the duplex and triplex DNA data as additional information. The osmotic-pressure induced mesophase transitions for duplex DNA in the presence of monovalent NaCl has been observed recently^22^, while in this paper we additionally present the duplex DNA mesophase transition data in the presence of monovalent KCl. Furthermore, the triplex mesophase transition was not measured elsewhere. Although the main focus of this work is to present and discuss the mesophase behavior of the G-quadruplex structures, we also present duplex DNA data (in the presence of KCl) and the triplex mesophase transition that was measured the for the first time. We then compare the observed G-quadruplex DNA mesophases with duplex and triplex DNA mesophases. We used “long” wild-type DNA (~1 micron long) in the duplex DNA measurements. See below for details on the triplexes.

### GMP-quadruplex

**Figure 3:**
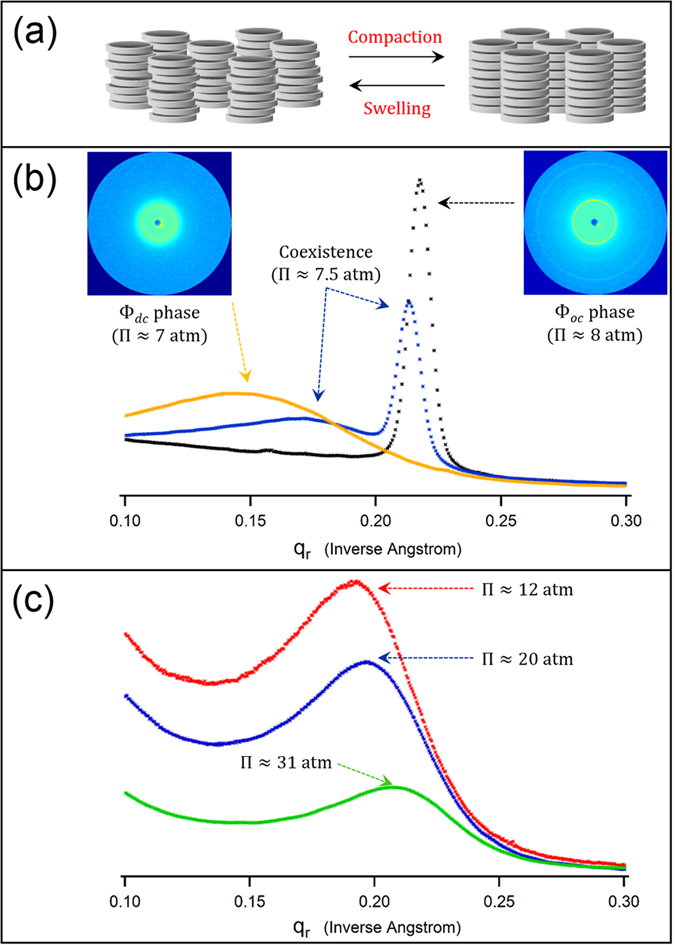
(**a**) Schematic illustration of the hexagonal columnar liquid-crystalline phases of G-quartets: In the Φ_*dc*_ phase (left), G-quartets in each column are displaced laterally around average columnar axes. In the Φ_*oc*_ phase (right), both G-quartet correlations within each column and column-column correlations are long-range. At the Φ_*dc*_ → Φ_*oc*_ transition, intercolumnar spacings between the neighboring columns (*d*_*int*_) decrease by 6.5–7 Å. (**b**) 1D intensity profiles (i.e., x-ray scattering intensities vs. momentum transfer in the radial direction) from *GMP-quadruplex* arrays at [KCl] = 0.3 M, showing the Φ_*dc*_ → Φ_*oc*_ transition: Essentially similar patterns were obtained with the intermolecular quadruplex (Fig. 2a), duplex (Fig. 2b), and triplex (Fig. 2c) (see *SI Appendix*). In the course of the Φ_*dc*_ → Φ_*oc*_ transition, the shape of the diffraction peak changes dramatically while the peak center is shifted to a higher *q*_*r*_ value. Two distinct types of peaks in the intensity profile coexist over a small range of Π, i.e., a phase-coexistence region. The procedure for x-ray diffraction peak fits is described in *SI Appendix*. **Insets:** X-ray images of the *GMP-quadruplex* arrays in the Φ_*dc*_ (left) and Φ_*oc*_ (right) phases. Higher order diffraction rings in the x-ray images of the Φ_*oc*_ phase confirm hexagonal packing (see *SI Appendix*). (**c**) 1D intensity profiles from *22-mer HT-quadruplex* (Fig. 1a,b) arrays at [KCl] = 0.3 M.

**Figure 4:**
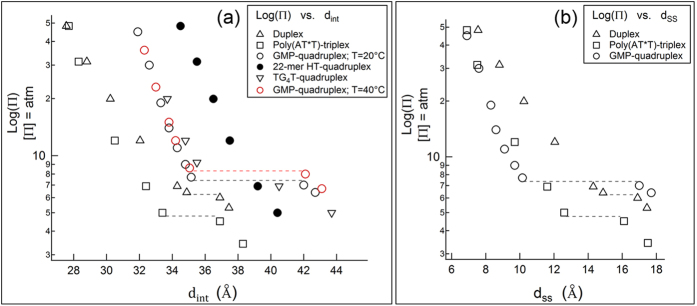
(**a**) Osmotic pressure vs. intercolumnar (interaxial) spacings (*d*_*int*_) for all structures that are shown in Figs 1 and 2: Duplex (triangle), *Poly*(AT*T)*-triplex* (square), *GMP-quadruplex* (circle), *22-mer HT-quadruplex* (filled circle), *TG*_4_*T-quadruplex* (inverted triangle). Horizontal lines show the Φ_*dc*_ → Φ_*oc*_ transitions. The lines are drawn approximately between the two *d*_*int*_ values determined from the centers of the coexisting two peaks in the phase-coexistence region (see Fig. 3). *Poly*(AT*T)*-triplex* measurements are performed under [MgCl] = 5 mM and [KCl] = 0.3 M. The measurements with other DNA structures are at [KCl] = 0.3 M in the absence of any other ions. The effect of temperature is shown only for *GMP-quadruplex* (where black and red symbols show the measurements at 20 and 40 °C, respectively, when the osmotic pressures are corrected for temperature). The slight temperature dependence of the transition pressure for the *GMP-quadruplex* is discussed in the text. For other structures data at T = 20 °C are shown only. **(b)** Osmotic pressure vs. surface-to-surface separation (*d*_*ss*_) for duplex, *Poly*(AT*T)*-triplex*, and *GMP-quadruplex* DNA. Here, *d*_*ss*_ = *d*_*int*_ − 2*a*, where *a* is the molecular (or columnar) radius ≈10 Å, 10.4 Å, 12.5 Å for duplex^26^, *Poly*(AT*T)*-triplex*^32^, and *GMP-quadruplex*^13^, respectively.

Increasing order continuously with increasing osmotic compression in the disordered columnar mesophase (Φ_*dc*_) is followed by a sudden collapse into the ordered columnar mesophase (Φ_*oc*_) with remarkable changes in the intercolumnar distance (*d*_*int*_) and the radial order (Fig. 3b). At the Φ_*dc*_ → Φ_*oc*_ transition of the *GMP-quadruplex*, in particular, the change in the intercolumnar distance (Δ*d*_*int*_) is about 6.5–7 Å (Fig. 4a). This change in the packing density corresponds to about 0.3–0.4 nm^3^ volume change per GMP nucleotide. It occurs concurrently with a significant lowering of the packing disorder. The radial disorder in the Φ_*dc*_ phase, due to lateral displacements of loosely stacked G-quartets around the mean columnar axis, is evident in the Gaussian-shaped broad diffraction radial intensity peaks. By comparison, the Lorentzian peak shape in the Φ_*oc*_ phase attests to the long-range nature of positional order. The correlation length in the ordered phase (equal to the inverse of the full-width-at-half-maximum of a Lorentzian function fitted to the x-ray diffraction radial intensity peak) is 5-to-6 neighbor separations for duplex DNA and 9-to-10 neighbor separations for *GMP-quadruplex*.

The slight temperature sensitivity of the Φ_*dc*_ → Φ_*oc*_ transition is shown for *GMP-quadruplex* (open circles in Fig. 4a). Temperature has no detectable effect on the packing density in the Φ_*oc*_ phase. The transition osmotic pressure (Π_*tr*_) increases by about 1 atm upon increasing temperature from 20 to 40 °C. The effects of temperature on Π_*tr*_ (as well as the effect of temperature on the DNA density at a fixed osmotic pressure Π) are appreciably smaller for other DNA structures and not shown. The transition for the *GMP-quadruplex* system is fairly sharp, and the integrated diffraction intensity (area under the diffraction peak) does not change through the transition. The integrated diffraction intensities from the same sample in the Φ_*dc*_ and Φ_*oc*_ phases are nearly the same (Fig. 3b). Phase-coexistence is pronounced over a significantly narrower range of osmotic pressures in the transitions of the duplex and *GMP-quadruplex* than in the transitions of the other structures. In particular, for *GMP-quadruplex*, the width of the phase-coexistence region is about 0.5 atm osmotic pressure.

### TG_4_T-quadruplex

When we followed the same procedure with the intermolecular *TG*_4_*T-quadruplex*, we observed first-order transitions similar to the Φ_*dc*_ → Φ_*oc*_ transition of the *GMP-quadruplex*. This suggests an abrupt change in the columnar organization of the short intermolecular *TG*_4_*T-quadruplex*, similar to the change in the columnar organization of the G-quartets at the Φ_*dc*_ → Φ_*oc*_ transition of the *GMP-quadruplex*. When this change in order is tuned by varying the osmotic pressure, we observe a much broader coexistence-region in the Φ_*dc*_ → Φ_*oc*_ transition for *TG*_4_*T-quadruplex* than for the *GMP-quadruplex*. For clarity, in Fig. 4a we show the intercolumnar spacings for the *TG*_4_*T-quadruplex* only at the upper and lower boundaries of the coexistence-region (inverted triangles). Intercolumnar spacings are about the same in the Φ_*oc*_ phases of the *TG*_4_*T-quadruplex* and *GMP-quadruplex* arrays under the same pressure.

### 22-mer HT-quadruplex

With the aim of obtaining ordered columnar phases of the *22-mer HT-quadruplex* arrays (similar to the Φ_*oc*_ phase of simpler model *GMP-quadruplex*), we increased the osmotic pressure compressing the disordered arrays of *22-mer HT-quadruplex* and measured their x-ray diffraction patterns. The radial packing order increases with increasing osmotic compression. However, the very sharp changes observed in the radial intensity profiles at the first-order Φ_*dc*_ → Φ_*oc*_ transition of the *GMP-quadruplex* are not seen in the radial intensity profiles of *22-mer HT-quadruplex* arrays (Fig. 3c).

**Figure 5:**
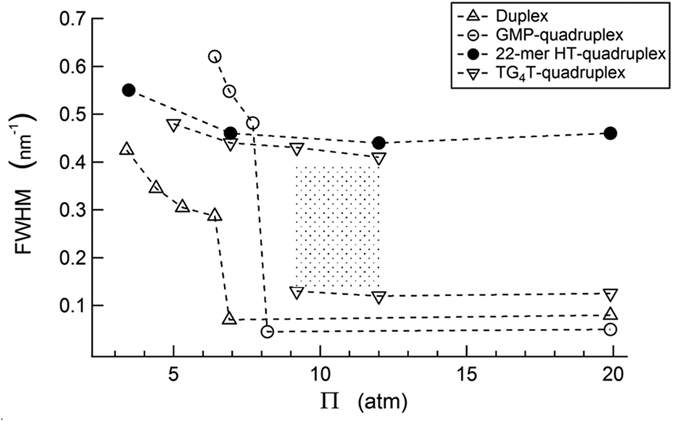
FWHM of the x-ray diffraction radial intensity peaks from the arrays of DNA structures: Symbols and structures are the same as in Fig. 4a,b. In the less-ordered phases (Φ_*dc*_), packing disorder decreases with increasing osmotic pressure for all structures. Further compression leads to Φ_*dc*_ → Φ_*oc*_ transitions with discontinuous changes in the packing order in duplex and *GMP-quadruplex* arrays, which occur concurrently with discontinuous changes in the packing densities (shown in Fig. 4a,b). The latter is not observed in the *22-mer HT-quadruplex* arrays (discussed in the text). Phase-coexistence is shown only for the *TG*_4_*T-quadruplex* (shaded area). Phase-coexistence is observed over significantly narrower ranges of osmotic pressures for the duplex and *GMP-quadruplex* compared with the *TG*_4_*T-quadruplex*. The error in the determination of FWHM is as big as ≈0.1 nm^−1^ in the case of broad Gaussian peaks (in the disordered phases). The errors in FWHM are smaller for the sharp Lorentzian peaks in the ordered phases (see *SI Appendix*).

When equilibrated under osmotic pressures less than the Φ_*dc*_ → Φ_*oc*_ transition osmotic pressure of the *GMP-quadruplex*, the measured positional disorder in *22-mer HT-quadruplex* array is smaller than the disorder in *GMP-quadruplex* array at the same pressure (Fig. 5), which can be argued to be a consequence of the constrained lateral motion of the G-quartets, connected by the sugar-phosphate backbone. This connectivity increases the stability of *22-mer HT-quadruplex* relative to the *GMP-quadruplex* and decreases its positional as well as stacking disorder. This is obviously true despite the TTA loops of the *22-mer HT-quadruplex* that extend laterally from the core G-quartets^5^, and could be conceived as promoting and not suppressing the disorder of the columns. However, when equilibrated under osmotic pressures greater than that of the Φ_*dc*_ → Φ_*oc*_ transition of *GMP-quadruplex, d*_*int*_ is 2–3 Å bigger for *22-mer HT-quadruplex* than for *GMP-quadruplex* at the same pressure (Fig. 4a). Under these conditions, the radial disorder in the *22-mer HT-quadruplex* array remains almost the same as in the disordered phase, while that of the *GMP-quadruplex* array drops significantly (Fig. 5, blue circles), possibly signaling the reverse action of laterally extended TTA loops in this case, amounting to a simple increase of the effective diameter of the columns.

### Poly(AT^*^T)-triplex

*Poly*(AT*T)*-triplex* samples are prepared in the presence of 5 mM Mg^2+^. The role of Mg^2+^ in the stability of DNA triplexes has been investigated^24^, and stable *Poly*(AT*T)*-triplex* at [Mg^2+^] = 5 mM is reported. Under sufficient osmotic pressures in presence of 5 mM Mg^2+^, *Poly*(AT*T)*-triplexes* self assemble into columnar aggregates (see *Methods*). Addition of 0.3 M K^+^, while keeping [Mg^2+^] = 5 mM, causes the following changes in the *Poly*(AT*T)*-triplex* arrays (under fixed pressure): (i) in the Φ_*oc*_ phase, expansion in the lateral direction (see *SI Appendix*) and (ii) in the Φ_*dc*_ phase, destabilization of the *Poly*(AT*T)*-triplex*. The osmotic pressure required to prevent the destabilization of the *Poly*(AT*T)*-triplex* depends on the concentrations of K^+^ and Mg^2+^. At the Φ_*dc*_ → Φ_*oc*_ mesophase transition of the *Poly*(AT*T)*-triplex* (in the presence of 0.3 M K^+^ and 5 mM Mg^2+^) *d*_*int*_ changes from about 37 Å to 33 Å (Fig. 4a). The changes were reversible for *d*_*int*_ less than ≈38 Å. At larger spacings, once the *Poly*(AT*T)*-triplexes* disassociate, the triplex formation cannot be reestablished by simply increasing the osmotic pressure. Thus, the *d*_*int*_ values for the *Poly*(AT*T)*-triplex* that we report in Table 1 are near the biggest distances where the transition can be observed at [K^+^] = 0.3 M.

## Discussion

Fiber diffraction data provide substantive evidence of helical stacking of the G-quartets^13^ in *GMP-quadruplex*, as well as helical stacking of the base-pairs^26^ in duplex DNA, at relative humidities corresponding to the osmotic pressures produced by the bathing PEG solutions that induced Φ_*oc*_ phases of these structures. These helical details are expected to be pronounced only in the presence of strong correlations between repeating units (base-pair for duplex and G-quartet for *GMP-quadruplex*) along the columnar axes. Based on this, one can argue that the helical nature of the base-stacking in DNA structures plays a key role in the Φ_*dc*_ → Φ_*oc*_ transitions of the DNA arrays. Transitions occur when the intercolumnar spacings (*d*_*int*_) are comparable to the helical pitch length (P), i.e., the axial distance per helical turn along the columnar axis (see Table 1). This implies that the formation of long-range translational and helical order along the columnar axis leads to long-range intercolumnar order in the Φ_*oc*_ phase. Additionally, the strength of the attraction, as seen in the volume change per nucleotide (Δ*V*_*pn*_) in Table 1, increases with increasing number of strands in the structure. Counterintuitively, the attraction induced at the transition increases with increasing linear or surface charge density (*λ* and *σ*, respectively). This scenario has been elaborated^27^,^28^ theoretically but is difficult to corroborate experimentally.

We examined the Φ_*dc*_ → Φ_*oc*_ mesophase transitions of duplex, triplex, and quadruplex DNA structures. In particular, the spontaneous formations of highly-ordered G-quadruplex columns (*GMP-quadruplex* and *TG*_4_*T-quadruplex*) under biologically relevant molecular crowding conditions are significant for their analogies with the stacking organization of G-quadruplex structures in the human telomere. A quasi-continuous helix that runs along the columnar axis by the stacking of G-quartets within the *TG*_4_*T-quadruplex* columns^20^ (similar to the helical stacking of the G-quartets in *GMP-quadruplex*) is possible with azimuthal rotations and arrangements of the *TG*_4_*T-quadruplex blocks* relative to the adjacent *blocks*.

The formation of uniaxially ordered columnar liquid crystals has been observed also in the case of the stacking of very-short-fragment (6 base-pairs long) DNA duplexes^25^. This stacking behavior was explained by the end-to-end adhesion of the exposed hydrophobic cores of the short DNA segments. From the observed Φ_*dc*_ → Φ_*oc*_ first-order mesophase transition of the *TG*_4_*T-quadruplex* arrays, as seen in the discontinuous change in the x-ray diffraction patterns tuned by varying molecular crowding conditions, it is possible to argue that the high-planarity of the G-quartets in the *TG*_4_*T-quadruplex* blocks makes the end-to-end stacking favorable. However, one could claim that the presence of the thymine bases at the ends of *TG*_4_*T-quadruplex* blocks would likely weaken the stacking interactions and in turn make the uniaxial helical ordering unfavorable. The broad range of osmotic pressures where the phase coexistence is observed in *TG*_4_*T-quadruplex* arrays (Fig. 5) might be attributed to the increased disorder due to the thymine bases at the ends.

In order to investigate the formation of ordered G-quadruplex columns in the telomere, the *22-mer HT-quadruplex* was specifically chosen for two reasons: (i) the four-repeat sequence AG_3_(TTAG_3_)_3_ is the likely candidate for the G-quadruplex formation *in vivo* and (ii) G-quadruplex conformation of this sequence under molecular crowding conditions is known. However, to the best of our knowledge, there is no direct evidence that the *22-mer HT-quadruplex* blocks would be organized into a columnar assembly in the non-crystalline state. Our osmotic stress experiments were designed to detect any sudden changes in the packing density and order, similar to the observed first-order mesophase transitions of the other model G-quadruplexes (*GMP-quadruplex* and *TG*_4_*T-quadruplex*). However, at all osmotic pressures *22-mer HT-quadruplex* blocks (when they are *unlinked*) were observed to make only disordered columns, characterized by almost unchanged radial disorder (Fig. 5), implying also a pronounced stacking disorder. At large osmotic pressures it appears as if the disordered columns would have an effective diameter augmented by the contribution of the dangling TTA loops, while at small osmotic pressures the phosphate backbone connectivity of the *22-mer HT-quadruplex* blocks enhances their stability. The robust disordered columnar assembly would possibly be the outcome of the attenuated stacking interactions between the G-quartet cores of the *22-mer HT-quadruplex* blocks, compared with the very pronounced stacking interactions within the highly-ordered columnar phases of the *GMP-quadruplex* and the *TG*_4_*T-quadruplex*. This preserves the fluid-like order in the *22-mer HT-quadruplex* columns at all crowding conditions. In fact, this could well be important in the *in vivo* context.

Another feature of the ordered phases of the DNA structures is revealed by comparison of the characteristic decay lengths in the Π vs. *d*_*int*_ curves (Fig. 4a) of duplex, triplex, and *GMP-quadruplex* DNA. On theoretical grounds one can quantify the intercolumnar distance dependence of the osmotic pressure of the array in terms of a short-range hydration characteristic length and a longer-ranged Debye screening length^29^. This approach describes the hydration and the electrostatic components of the total interaction between the columnar structures in an ordered array. In the case of duplex DNA in monovalent salt solutions, at large separations in the ordered phase, the apparent decay length from the Π vs. *d*_*int*_ curve is close to the Debye length, suggesting that the electrostatic interactions dominate at these separations^22^. When the surface-to-surface separation is smaller than about 7–8 Å, the Π vs. *d*_*int*_ curves for all ionic concentrations converge to a single curve, suggesting also a universal short-range hydration repulsion that is independent of ionic strength (for details see ref. 22). Similarly, triplex DNA exhibits the same interaction regimes, with comparable Debye lengths at large interaxial separations, except that the hydration decay length in the high density regime is smaller than in the case of duplex DNA. In this respect the triplex DNA lies in between the behavior exhibited by the duplex DNA and the G-quadruplexes. In fact, in the case of G-quadruplexes, neither the Debye length nor the expected characteristic length for the short-range hydration interactions (on the order of the size of a water molecule) are as anomalously small as the apparent lengths reported here (~1 Å). The nature of these extremely short range interactions observed in G-quadruplex arrays thus remains to be elucidated.

As a side note, the radial disorder in the Φ_*dc*_ phase is more pronounced in the *GMP-quadruplex* arrays than in the duplex, *22-mer HT-quadruplex*, and *TG*_4_*T-quadruplex* arrays (Fig. 5). In the disordered phases, the repeating units along the columnar (or molecular) axes are constrained by (i) the relatively weak base-stacking interactions and (ii) the sugar-phosphate links between the adjacent units. The data in Fig. 5 point to the increased molecular stability by the sugar-phosphate backbone in duplex, *22-mer HT-quadruplex*, and *TG*_4_*T-quadruplex* relative to the *GMP-quadruplex*. Nonetheless, the base-stacking interactions between the G-quartets are obviously strong enough and lead to the formation of disordered *GMP-quadruplex* columns at osmotic pressures as low as ≈3–4 atm (corresponding to ≈15 wt% PEG 8000 concentration).

Molecular crowding - as quantified by its proxy, the PEG solution osmotic pressure - certainly plays an important role in the stabilization of different DNA structures and regulation of their respective functions^10^. By explicitly showing and analyzing how the solution PEG osmotic pressure controls the DNA density, the related molecular order, and the phase transitions between differently ordered dense DNA-analogue arrays, we can take an additional step in understanding how the complicated *in vivo* environment regulates different functionalities of nucleic acids.

## Methods

### Sample preparation

DNA oligonucleotide samples were ordered from Integrated DNA Technologies and were stored in a freezer until the time of measurements. Below we describe the methods of preparations of each DNA structure considered in this manuscript.

#### GMP-quadruplex

We prepared the GMP solutions (1 mg/ml) at [KCl] = 0.3 M with stirring at room temperature for about 2–3 hours. We then mixed 1 ml samples of the prepared GMP-solution with 4 ml 25 wt% PEG 8000 solutions (containing 0.3 M K^+^), i.e., the final solution contains ~1 mg of GMP under 20 wt% PEG 8000 and 0.3 M K^+^. Under these conditions, GMP precipitates and pellets are formed by centrifugation. We transferred the collected pellets into new PEG 8000 solutions (at various wt% concentrations) for the x-ray diffraction experiments.

#### TG_4_T-quadruplex

Quadruplex formation was induced by heating TG_4_T oligonucleotide solution at 80 °C for 5 min and then cooling to room temperature at [KCl] = 0.1 M. The oligonucleotide concentration in the annealing solution was ~0.1 mg/ml. *TG*_4_*T-quadruplex* is extensively studied in the literature. Their conformation in K^+^ solutions is well-known^30^. We measured the CD spectra of the TG_4_T solutions before and after the heat incubation to confirm the parallel-stranded intermolecular *TG*_4_*T-quadruplex* formation (see *SI Appendix*).

#### 22-mer HT-quadruplex

Quadruplexes were formed by heating the four-repeat telomeric sequence AG_3_(TTAG_3_)_3_ solution at 95 °C and [KCl] = 50 mM for 5 min and then cooling to room temperature. The oligonucleotide concentration in the annealing solution was ~0.1 mg/ml. We measured the CD spectra of the samples to ensure the formation of the structure (see *SI Appendix*). We then equilibrated the quadruplexes in PEG 8000 solutions (40 wt%). The structural conversion of the AG_3_(TTAG_3_)_3_ sequence to parallel-stranded *22-mer HT-quadruplex* conformation in PEG solutions was seen^6^,^7^. *22-mer HT-quadruplex* arrays aggregated in the 40 wt% PEG 8000 solutions were collected by centrifugation. The concentrated pellet was then transferred into new solutions of PEG 8000 (at various wt% concentrations) and [KCl] = 0.3 M for equilibration (~48 hours). Before the x-ray diffraction experiments, the solutions were centrifuged for long hours (~20–30 hours) at 4 °C and 30,000 g. The pellets (~1 mg of weight) were held under the same solution conditions (0.3 M K^+^ and the desired PEG wt%) during the diffraction measurements.

#### Poly(AT*T)-triplex

*Poly*(AT*T)*-triplex* structures are made of 50 bases long Poly(A) and Poly(T) sequences, by heating the solutions of Poly(A) and Poly(T) (mixed at 1:2 ratio) to 90 °C and slowly cooling to room temperature. The oligonucleotide concentration in the annealing solution (containing 5 mM Mg^2+^) was ~0.1 mg/ml. Following the annealing, we measured the CD spectra of the samples to ensure that the *Poly*(AT*T)*-triplexes* were formed (see *SI Appendix*). We then concentrated the *Poly*(AT*T)*-triplex* solution to about 1 mg/ml oligonucleotide concentration. The *Poly*(AT*T)*-triplex* arrays are formed in PEG 8000 solutions: We mixed 1 ml samples of the 1 mg/ml *Poly*(AT*T)*-triplex* solution with 1 ml 50 wt% PEG 8000 (both containing 5 mM Mg^2+^). Thus the final solution contained ~1 mg of *Poly*(AT*T)*-triplex* under 25 wt% PEG 8000. Self-aggregated *Poly*(AT*T)*-triplex* arrays are equilibrated in the 25 wt% PEG 8000 solutions for about 48 hours and then collected by centrifuging for ~20 hours at 4 °C and 30,000 g. We then transferred the collected pellets into large volumes (~5 ml) of PEG 8000 bathing solutions at various wt% concentrations (also containing the desired ionic conditions, i.e., 5 mM Mg^2+^ and 0.3 M K^+^). We performed the x-ray diffraction measurements after equilibrating the *Poly*(AT*T)*-triplex* arrays in the bathing solutions for about 48 hours.

### Data collection

X-ray diffraction measurements are made using the in-house setup at the UMass Amherst Physics Department. Brief explanations of the x-ray diffraction data analysis are given in the caption to Fig. 3. See also *SI Appendix* for more details. CD spectra measurements are carried out in the School of Medicine at Case Western Reserve University.

### Osmotic pressure data

Temperature-dependent osmotic pressure data of PEG (molecular weight 8000 Daltons) solutions are from ref. 11. The osmotic pressure of PEG, as well as the temperature dependence of the osmotic pressure of PEG, are not new. They have been reproduced by a variety of experimental methods (e.g., vapor pressure osmometer, membrane osmometer).

## Additional Information

**How to cite this article**: Yasar, S. *et al*. X-ray characterization of mesophases of human telomeric G-quadruplexes and other DNA analogues. *Sci. Rep.* **6**, 27079; doi: 10.1038/srep27079 (2016).

## Supplementary Material

Supplementary Information

## Figures and Tables

**Figure 1:**
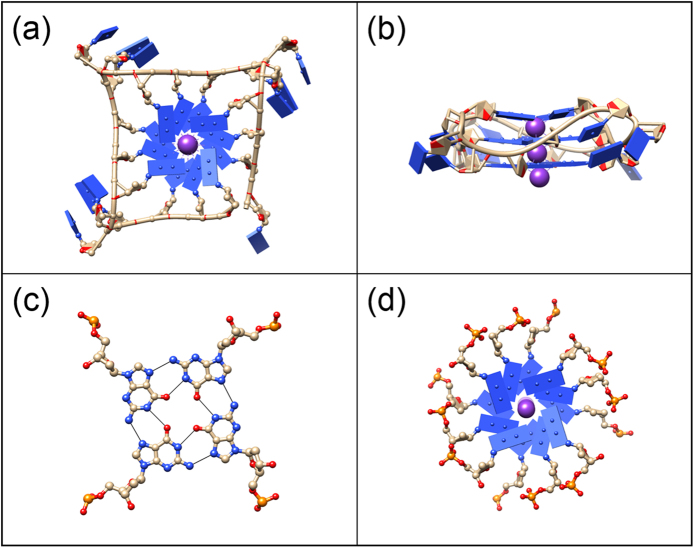
Schematic representations of the structures. (**a**) Top view of the parallel-stranded *22-mer HT-quadruplex* (PDB: 1KF1) formed by the sequence AG_3_(TTAG_3_)_3_. In this conformation (observed in the crystalline state^5^ as well as in the non-crystalline state under molecular crowding conditions^6^,^7^), four parallel GGG runs form a stack of three planar G-quartets in the central core and TTA segments fold into loops projecting outwards. (**b**) Side view of the *22-mer HT-quadruplex*. K^+^ ions (purple) reside inside the G-quadruplexes and they are positioned between the adjacent G-quartets. (**c**) G-quartet made of four GMP monomers. Thin black lines represent the hydrogen bonds holding nucleotides together in a planar cyclic arrangement. (**d**) Top view of the *GMP-quadruplex* in ordered columnar phase, where stacking is helical with an azimuthal rotation of 30° between adjacent G-quartets^13^. G-quartets in c & d are modeled from *22-mer HT-quadruplex* (PDB: 1KF1) shown in a & b.

**Figure 2:**
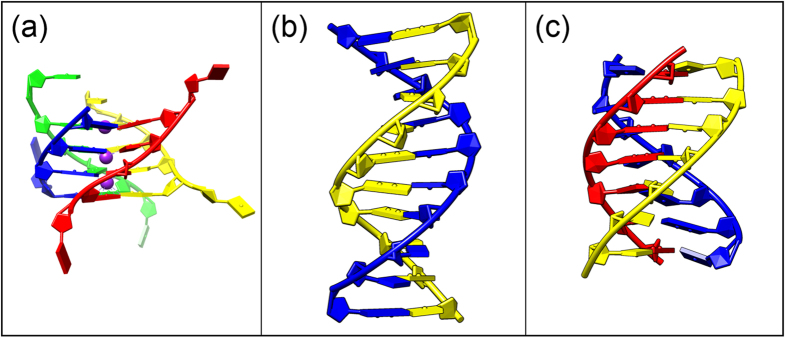
Schematic representations of the other DNA structures considered in this manuscript. (**a**) Side view of the intermolecular parallel-stranded *TG*_4_*T-quadruplex* (PDB: 244D) made of four *TG*_4_*T* oligonucleotides. In this quadruplex^20^, G-quartets are formed by hydrogen-bonding of the four parallel GGGG runs. Unlike the *GMP-quadruplex*, in the *TG*_4_*T-quadruplex*, G-quartets are connected via four sugar-phosphate strands. Similar to the other quadruplex structures (shown in Fig. 1), K^+^ ions (purple) reside inside the *TG*_4_*T-quadruplex*. (**b**) Side view of the duplex DNA in its B-form. (**c**) Side view of the triplex DNA (PDB: 134D). We used *Poly*(AT*T)*-triplex* made of 50 bases long Poly(A) and Poly(T) oligonucleotides^31^ in the presence of Mg^2+^ (explained in the text). In this figure, we used non-standard color-coding for the bases in order to differentiate between different strands of the structures.

**Table 1:** Quantitative information regarding the structure and the Φ_*dc*_ → Φ_*oc*_ transitions of duplex, *Poly*(AT*T)*-triplex*, and *GMP-quadruplex* DNA.

	***a*****(nm)**	***λ*****(e/nm)**	***σ*****(e/nm**^**2**^)	**P(nm)**	**Change in** ***d***_***int***_	**Δ*****d***_***int***_**(nm)**	**Δ*****A***_***cell***_**(nm**^**2**^)	**Δ*****V***_***pn***_**(nm**^**3**^)
Duplex	1	6	0.95	3.4	≈3.7-to-3.5 nm	0.19–0.21	0.11–0.12	0.19–0.20
*Poly*(AT*T)*-triplex*	1.04	9	1.38	3.4	≈3.7-to-3.3 nm	0.35–0.40	0.19–0.22	0.22–0.24
*GMP-quadruplex*	1.25	12	1.53	4.1	≈4.2-to-3.5 nm	0.65–0.70	0.43–0.46	0.36–0.39

The structural parameters, radius (*a*) and helical pitch length (P), are from refs 13,26 and 32.

The distances d_int_ are measured in 0.3 M K^+^ solutions for duplex and *GMP-quadruplex*. These distances for duplex and *GMP-quadruplex* decrease without a significant change in Δ*d*_*int*_ with increasing K^+^ concentration. The *Poly*(AT*T)*-triplex* measurements are carried out in the presence of 0.3 M K^+^ and 5 mM Mg^2+^. Lowering Mg^2+^ concentration any further (less than 5 mM), while keeping the K^+^ concentration fixed at 0.3 M, results in the disassociation of the triplexes (see *SI Appendix*).

*A*_*cell*_ is the hexagonal cross-sectional area surrounding the duplex, triplex, or *GMP-quadruplex*. Δ*A*_*cell*_ is the change in the *Wigner-Seitz cell* area at the transition.

Δ*V*_*pn*_ is the change in the volume per nucleotide at the transition. The change in the volume per stacking unit (i.e., base-pair for duplex, base-triplet for triplex, and G-quartet for *GMP-quadruplex*) is equal to Δ*A*_*cell*_ multiplied by the base-stacking height (see *SI Appendix*). The overall uncertainty in the determination of Δ*V*_*pn*_ is about 10%.
