# Supplementary material for: X-ray characterization of mesophases of human telomeric G-quadruplexes and other DNA analogues
Source: Sci Rep. 2016 Jun 2;6:27079. doi: 10.1038/srep27079 (PMC4890033; doi:10.1038/srep27079)
Supplement: Supplementary Information [file srep27079-s1.pdf]

Supplementary Information for "X-ray characterization of mesophases of human telomeric G-quadruplexes and other DNA analogues"

Selcuk Yasar, Jacob B. Schimelman, M. Alphan Aksoyoglu, Nicole F. Steinmetz, Roger H. French, V. Adrian Parsegian & Rudolf Podgornik

S1 DNA structure characterization - CD spectra

In this section we describe the methods of characterizations of the *22-mer HT-quadruplex*, the TG_4T*-quadruplex*, and the *Poly(AT*T)-triplex*. We compare our obtained CD spectra of these DNA structures with spectra in the literature [1–4] and confirm their conformations in solution. The measured CD spectra are shown in Figure S1.

The annealing solution conditions for the *22-mer HT-quadruplex*, the TG_4T*-quadruplex*, and the *Poly(AT*T)-triplex* were described in detail in the *Methods* Section in our manuscript. Briefly here, *22-mer HT-quadruplexes* and TG_4T*-quadruplexes* were formed in the presence of KCl (50 mM and 100 mM, respectively) only. The annealing solution for the *Poly(AT*T)-triplexes* contained 5 mM $MgCl_2$ only. Oligonucleotide concentrations in the annealing solutions were fixed at ∼0.1 mg/ml for all the structures. The CD spectra are measured following the annealing.

The samples of the triplex and quadruplex DNA structures that we prepared at the UMass Amherst Physics Department (using the protocols described in *Methods* in the manuscript) were sent to the School of Medicine at Case Western Reserve University, where CD spectra measurements were performed using the Circular Dichrosim Spectrometer (AVIV) for DNA-structure characterization (see Figure S1). X-ray diffraction measurements were carried out at the UMass Amherst Physics Department. Following annealing, the triplex and quadruplex DNA structures were transferred into various solutions with the desired ionic and molecular crowding conditions (see *Methods* in the manuscript) for the x-ray diffraction experiments.

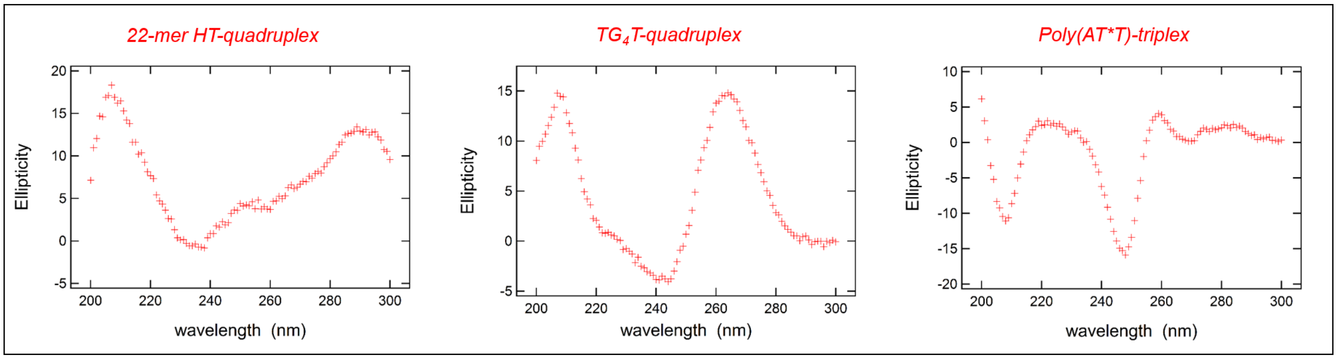

Figure S1: Measured CD spectra of the *22-mer HT-quadruplex* (left), TG_4T*-quadruplex* (middle), and *Poly(AT*T)-triplex* (right). The oligonucleotide concentration was ∼0.1 mg/ml for all the structures. For the ionic conditions, see above as well as the *Methods* in the manuscript.

S2 X-ray diffraction data analysis

X-ray diffraction measurements are made using the in-house setup at the UMass Amherst Physics Department (see Section S3). Brief explanations of the x-ray diffraction data analysis are given in the caption to Figure 3. In this section we describe the obtained radial intensity distributions and their fittings in detail.

For data centering and extracting 1D diffraction intensity distributions from 2D images, *SAXSGUI* (from Rigaku) is used. Intensity profiles (shown in a linear scale in Figure S2) are calculated by radial integration of the intensity distributions in the 2D raw x-ray diffraction images. The radial component of the scattering wave vector (q_r) is calculated from

$$q_r=(4\pi/\lambda)sin(\theta/2) \ , \tag{S1}$$

where θ is scattering angle and λ is x-ray wavelength. The interaxial spacings (d_{int}) are determined from the peak positions (q_0) and assumed hexagonal packing symmetry as

$$d_{int}=\left(2/\sqrt{3}\right)d_{Bragg} \ , \tag{S2}$$

where $d_{Bragg}=2\pi/q_0$.

Sharp diffraction peaks in the condensed phases are fitted to a Lorentzian convolved with a Gaussian (in order to take into account instrumental broadening) after background subtraction (Figure S3). The Gaussian and the Lorentzian are described as

$$G(q,q_0,\sigma_G) = A \times Exp\left[-\frac{1}{2}\left(\frac{q-q_0}{\sigma_G}\right)^2\right] \tag{S3}$$

and

$$L(q,q_0,\sigma_L) = \frac{B}{(q-q_0)^2+\sigma_L^2} \tag{S4}$$

respectively.

In Figure S3 we illustrate the fits of the ordered columnar phase diffraction peak to a Gaussian, a Lorentzian, and the convolution of Gaussian with a Lorentzian. The latter is called the Voigt function,

$$V(q,q_0,\sigma_L) = G(q,q_0,\sigma_G) * L(q,q_0,\sigma_L) \ . \tag{S5}$$

The diffraction peaks in the ordered columnar DNA phases are described best by the Voigt function when $\sigma_G = 0.004Å^{-1}$ (see also Section S3 for the determination of instrumental broadening from the analysis of the direct x-ray beam shape.).

Broad diffraction peaks in the disordered columnar DNA phases are Gaussian-shaped and fitted to $G(q,q_0,\sigma_G)$. The radial disorder in the disordered columnar DNA arrays leads to Gaussian broadening of the diffraction peaks around average momentum transfer q_0 (which is related to the average distance between the molecules). In this case we take into account the effect of instrumental broadening by subtracting the $0.004Å^{-1}$ from the σ_G value found from the fit (see Figure S3).

The FWHM given in Figure 5 in the manuscript are then calculated as

$$\text{FWHM} = 2\sqrt{2\ln 2}\ \sigma_G \tag{S6}$$

and

$$\text{FWHM} = 2\ \sigma_L \tag{S7}$$

for the disordered and the ordered columnar DNA phases, respectively.

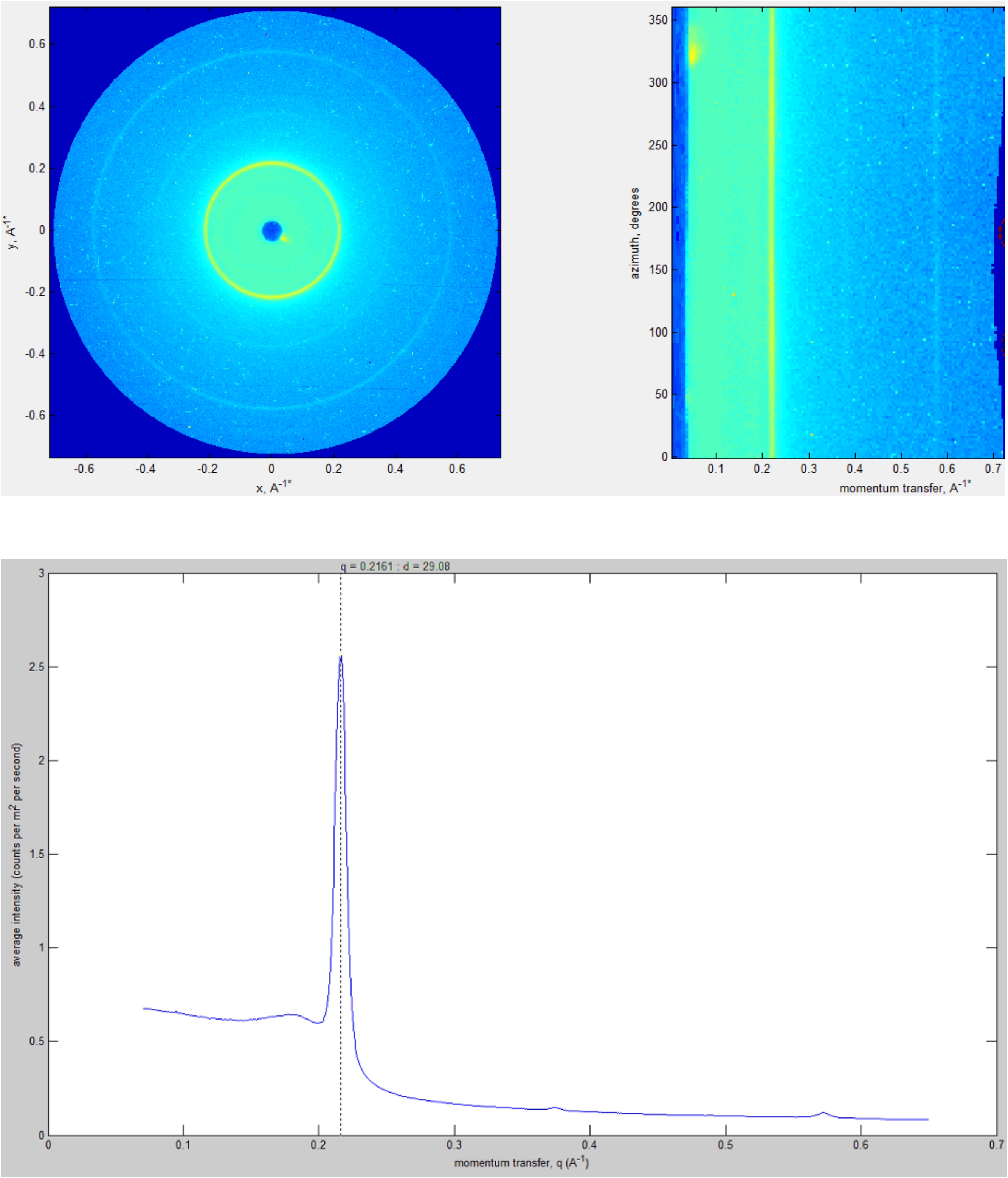

Figure S2: **Top left:** Typical 2D raw x-ray image obtained from *GMP-quadruplex* samples using in-house setup at the UMass Amherst Physics Department (see Section S3). **Top right:** Azimuthal angle vs. radial component of the momentum transfer (q_r) obtained using *SAXSGUI* software. **Bottom:** Intensity vs. q_r obtained by radial integration. As shown here for the *GMP-quadruplex* samples, higher-order diffraction peaks confirm hexagonal packing. (For the evidence of hexagonal symmetry in duplex and *Poly(AT*T)-triplex* arrays in their ordered phases, see Refs. [5] and [6], respectively.) The broad Gaussian superimposed with the sharp Lorentzian first-order diffraction peak is due to phase-coexistence in this particular sample.

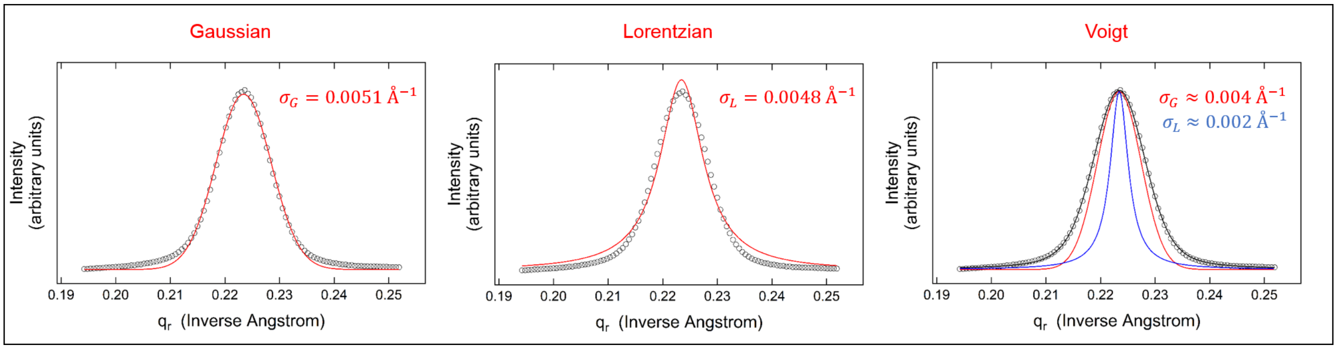

Figure S3: The fits of the ordered columnar DNA phase diffraction peaks to a Gaussian (left), a Lorentzian (middle), and the Voigt (right) function. The latter (convolution of a Gaussian and a Lorentzian) describes the shape of the peak. Here, the Gaussian (red in the right panel) is due to instrumental broadening. The Lorentzian (blue in the right panel) is due to the long-range nature of positional order. The correlation length in the ordered columnar phase is equal to the inverse of the FWHM of the Lorentzian, which is calculated using Eq. S7 above.

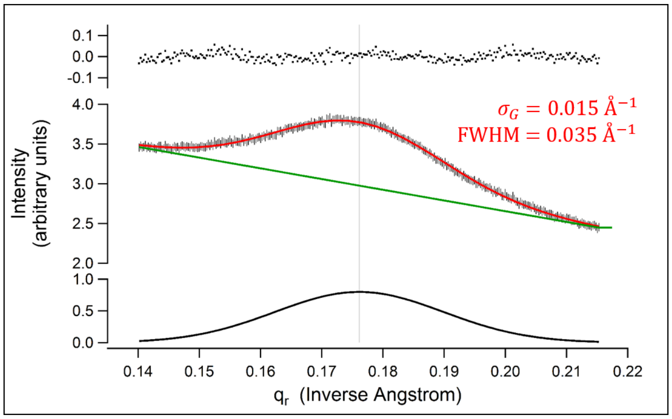

Figure S4: The fitting of the broad Gaussian-shaped x-ray diffraction peak in the fluctuating DNA mesophase. Gaussian broadening and the corresponding FWHM (given in Figure 5 in the manuscript), due to positional disorder in the hexagonal array, are calculated as $\sigma_G = 0.015$ Å^{-1} - 0.004 Å^{-1} $= 0.011$ Å^{-1} and FWHM $= 2\sqrt{2\ln 2}\ \sigma_G \approx 0.025$ Å^{-1}. We subtract 0.004Å^{-1} from σ_G in order to take into account instrumental broadening. See Figure S3 for the determination of the effect of instrumental broadening on the diffraction peak widths. See also Section S3 for the determination of instrumental broadening from the analysis of the direct x-ray beam shape.

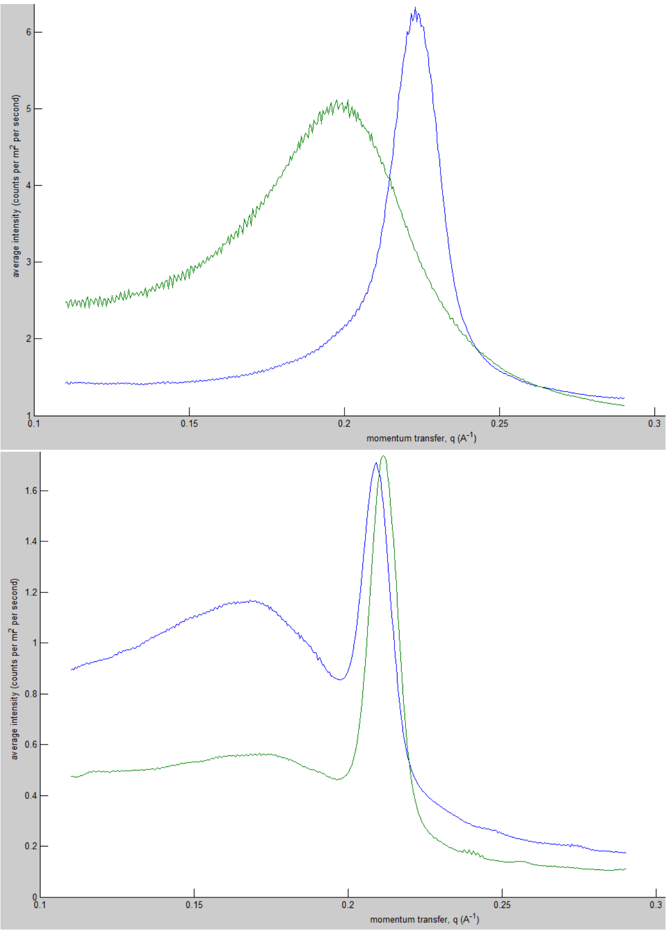

Figure S5: First-order radial intensity peaks for the *Poly(AT*T)-triplex* (top) and TG_4T*-quadruplex* (bottom) samples, showing the changes at the transition. In the top panel, green and blue (that are measured at the osmotic pressures $\Pi \approx 4$ atm and $\Pi \approx 5$ atm, respectively) correspond to the ordered and disordered phases. In the bottom panel, the osmotic pressures corresponding to the green and blue diffraction intensities are $\Pi \approx 9$ atm and $\Pi \approx 7$ atm, respectively. At these pressures, coexistence of the ordered and disordered phases are observed as explained in the main text. The ionic conditions are also described in the main text.

Finally, x-ray diffraction patterns in the ordered and disordered phases of the duplex, triplex, and the G-quadruplex structures considered in this manuscript are essentially similar. We have shown the first-order diffraction peaks for the *GMP-quadruplex* and *22-mer HT-quadruplex* in the main text (Figure 3-b & 3-c). Here in Figure S5, we show similar patterns for the *Poly(AT*T)-triplex* (top) and TG_4T*-quadruplex* (bottom), both in the ordered and disordered phases. Please see Ref. [7] for the documentation of similar diffraction patterns from duplex DNA as well as the changes observed at the ordering transitions of duplex DNA.

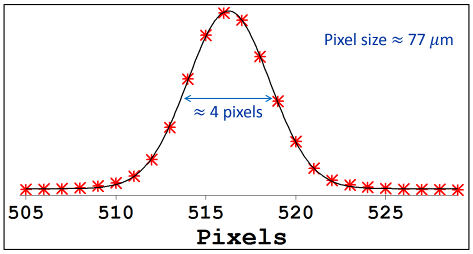

Figure S6: Radial intensity profile of the x-ray beam used in x-ray diffraction peak measurements, explained in Section S2: The width of the Gaussian describing the radial intensity distribution of the incident x-ray beam cross-section on the detector surface is ≈0.3 mm. This effect (called "instrumental broadening") is taken into account in the x-ray diffraction peak fittings and when calculating the standard deviation of the momentum transfer in the radial direction (q_r).

S3 X-ray diffraction setup

DNA pellets are transferred into sample cells for x-ray measurements and the sample cells are sealed against evaporation. MicroMax-002+ x-ray generator from Rigaku with a microfocus sealed tube x-ray source is used in the measurements. The optic module with multilayer reflectors is designed by Rigaku based on the need of our experiments. MicroMax-002+ combined with the optical system leads to a beam of monochromatic radiation (wavelength λ=1.54 Å and focus at ∼400 mm from the end of optic module). The x-ray slit from Huber Diffraktionstechnik is used to shield the background scatter and to control the size of the beam. After passing the slit, the beam of about 0.6 mm diameter enters the sample and detector chamber.

2D X-ray images are taken with a Rigaku Mercury-3 CCD X-ray detector (75 mm diameter circular image area and ∼75 μm pixel size). The sample-to-detector distance is ∼200 mm. The angular range of x-ray scattering covers a wave vector range from 0.05Å^{-1} to 0.75Å^{-1} in which q_r=$(4\pi/\lambda)sin(\theta/2)$, where θ is the scattering angle.

Samples are exposed to an x-ray beam having a well-defined steady Gaussian shape, and the resolution is increased by long data-collection times. In Figure S6 we show the cross-section of the incident x-ray beam used in this study. Additionally, in Section S2 we described the method to deconvolute the Gaussian beam function (determined from the beam shape) from the measured diffraction peaks to account for instrumental broadening.

The width of the Gaussian describing the radial intensity distribution of the incident x-ray beam cross-section on the detector surface is ≈0.3 mm, which corresponds to $\theta/2 \approx 0.3^\circ$ when the sample-to-detector distance is ∼220 mm. Using Eq. S1 and the x-ray wavelength $\lambda = 1.54$ Å, we find the corresponding broadening in the intensity vs. q_r diffraction peaks as

$$\sigma_{beam}=(4\pi/1.54\ \text{Å})sin(0.3^\circ) \approx 0.004\ \text{Å}^{-1}.$$

Sample preparation for the x-ray diffraction experiments: During the x-ray diffraction measurements, the samples were held in a vacuum-tight cells, enclosed between two thin mylar windows (see Figure S7). We prepared a sample cell for each PEG wt%, rather than sequential dilution.

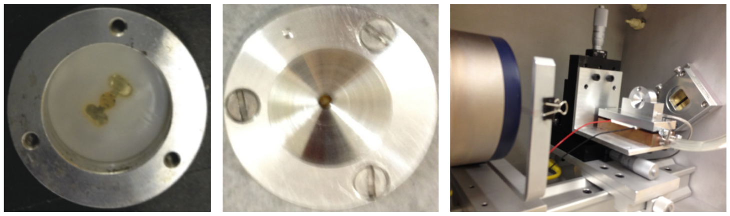

Figure S7: Left: Sample cell with DNA pellet and reservoir (i.e., solution with the desired ionic concentration and PEG wt%). Middle: The sample cell is secured with screws. Right: Temperature is controlled with a thermocouple and chiller-radiator (placed under the sample cell) during the x-ray diffraction measurements.

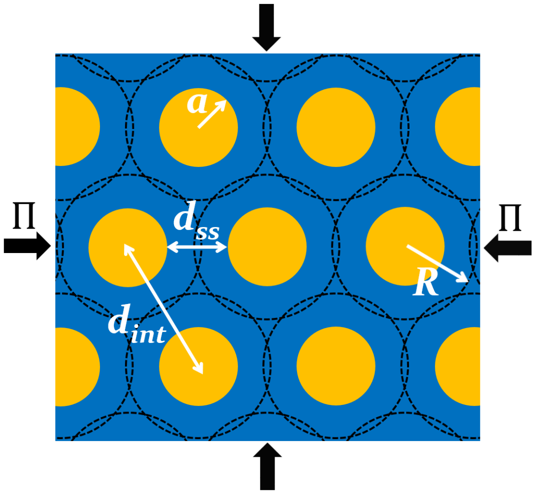

Figure S8: Cylindrical cell model: Dashed lines are the Wigner-Seitz cell boundaries when the cell radius is $R = 1.05(d_{int}/2)$ and $d_{ss} = d_{int} - 2a$, where a is the molecular (or columnar) radius.

S4 Cylindrical cell model

In a hexagonal array of long cylindrical molecules, the hexagonal cross-sectional area surrounding each molecule can be approximated by a circle of the same area known as the *Wigner-Seitz cell* [8]. The radius R of this cell is found by constructing a *Voronoi cell* around each molecule, calculating its cross-sectional area, equating this to πR^2, and solving for R. The result is:

$$R = \left(\frac{\sqrt{3}}{2\pi}\right)^{\frac{1}{2}} d_{int} \simeq 0.525\ d_{int} \simeq 1.05\ \frac{d_{int}}{2} \tag{S8}$$

where d_{int} is the interaxial distance, or the center-to-center separation, between nearest-neighbor molecules in the 2D hexagonal array (Figure S4).

We calculate the change in the Wigner-Seitz cell area (ΔA_{cell}) at the ordering transitions using the measured values of the smallest d_{int} in the disordered columnar phase and the biggest d_{int} in the ordered columnar phase. The change in d_{int} and the calculated ΔA_{cell} at the transition are given in Table 1 in the manuscript.

We calculate the change in the volume per nucleotide at the transition (ΔV_{pn}) as

$$\Delta V_{pn} = \mathrm{h}\ \frac{\Delta A_{cell}}{\mathrm{n}}\ , \tag{S9}$$

where h is the base-stacking height (=0.34 nm) and n is the number of nucleotides per repeating-unit along the molecule (n=2, 3, and 4 for duplex, triplex, and quadruplex structures, respectively). We assume that h is the same for all the DNA structures and does not change at the transition.

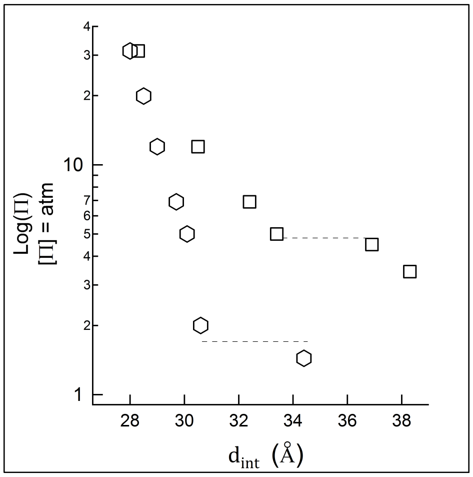

Figure S9: *Poly(AT*T)-triplex* mesophase transitions at two different bathing solution conditions. Hexagon: [MgCl]=5 mM only, [KCl]=0. Square: [MgCl]=5 mM and [KCl]=0.3 M.

S5 *Poly(AT*T)-triplex* mesophase transitions and stability in K^+ solutions

We prepared Poly(AT*T) DNA triplexes in the presence of Mg^{2+}. Triplex arrays are first formed and equilibrated for 48 hours in 25 wt% PEG 8000 solutions in the presence of 5 mM Mg^{2+} only. The measured ordering transition of the *Poly(AT*T)-triplex* is shown below in Figure S9.

When K^+ is added to the *Poly(AT*T)-triplex* solution while keeping the [Mg^{2+}] fixed, interaxial separation d_{int} increases in both mesophases. The osmotic pressure required for the ordering transition strongly depends on the [Mg^{2+}]/[K^+] ratio. In Figure S9 the transitions at [Mg^{2+}]=5 mM in the presence (0.3 M) and absence of KCl are displayed.
While the interaxial separation in the *Poly(AT*T)-triplex* arrays increases upon addition of K^+, the triplexes are also destabilized at larger separations. At [KCl]=0.3 M, *Poly(AT*T)-triplexes* disassociate when d_{int} is larger than 38Å. The changes in the triplex arrays at the mesophase transition are not reversible once the triplexes disassociate.

References

[1] Neidle, S. & Balasubramanian, S. *Quadruplex Nucleic Acids* (RSC Publishing, 2006).

[2] Vorlickova, M., et al. Circular dichroism and guanine quadruplexes. *Methods* **57,** 64-75 (2012).

[3] Morikawa, M., et al. *RSC Adv.* **3,** 25694 (2013).

[4] Qiu, X., Parsegian, V.A. & Rau, D.C. Divalent counterion-induced condensation of triple-strand DNA. *Proc. Natl. Acad. Sci. USA* **107,** 21482–21486 (2010).

[5] Durand, D., Doucet, J. & Livolant, F. A study of the structure of highly concentrated phases of DNA by x-ray diffraction. *J. Phys. II France* **2,** 1769-1783 (1992).

[6] Qiu, X., Parsegian, V.A. & Rau, D.C. Divalent counterion-induced condensation of triple-strand DNA. *Proc. Natl. Acad. Sci. USA* **107,** 21482–21486 (2010).

[7] Yasar, S., et al. Continuity of states between the cholesteric−line hexatic transition and the condensation transition in DNA solutions. *Scientific Reports*, **4,** 6877 (2014).

[8] Fuoss, R., Katchalsky, A. & Lifson, S. The potential of an infinite rod-like molecule and the distribution of the counterions. *Proc. Natl. Acad. Sci. USA* **37,** 579-589 (1951).
